# Push Force Analysis of Anchor Block of the Oil and Gas Pipeline in a Single-Slope Tunnel Based on the Energy Balance Method

**DOI:** 10.1371/journal.pone.0150964

**Published:** 2016-03-10

**Authors:** Yifei Yan, Lisong Zhang, Xiangzhen Yan

**Affiliations:** 1 College of Mechanical and Electronic Engineering, China University of Petroleum, Qingdao 266580, China; 2 College of Pipeline and Civil Engineering, China University of Petroleum, Qingdao 266580, China; Beijing University of Posts and Telecommunications, CHINA

## Abstract

In this paper, a single-slope tunnel pipeline was analysed considering the effects of vertical earth pressure, horizontal soil pressure, inner pressure, thermal expansion force and pipeline—soil friction. The concept of stagnation point for the pipeline was proposed. Considering the deformation compatibility condition of the pipeline elbow, the push force of anchor blocks of a single-slope tunnel pipeline was derived based on an energy method. Then, the theoretical formula for this force is thus generated. Using the analytical equation, the push force of the anchor block of an X80 large-diameter pipeline from the West—East Gas Transmission Project was determined. Meanwhile, to verify the results of the analytical method, and the finite element method, four categories of finite element codes were introduced to calculate the push force, including CAESARII, ANSYS, AutoPIPE and ALGOR. The results show that the analytical results agree well with the numerical results, and the maximum relative error is only 4.1%. Therefore, the results obtained with the analytical method can satisfy engineering requirements.

## 1. Introduction

**Fig 1:**
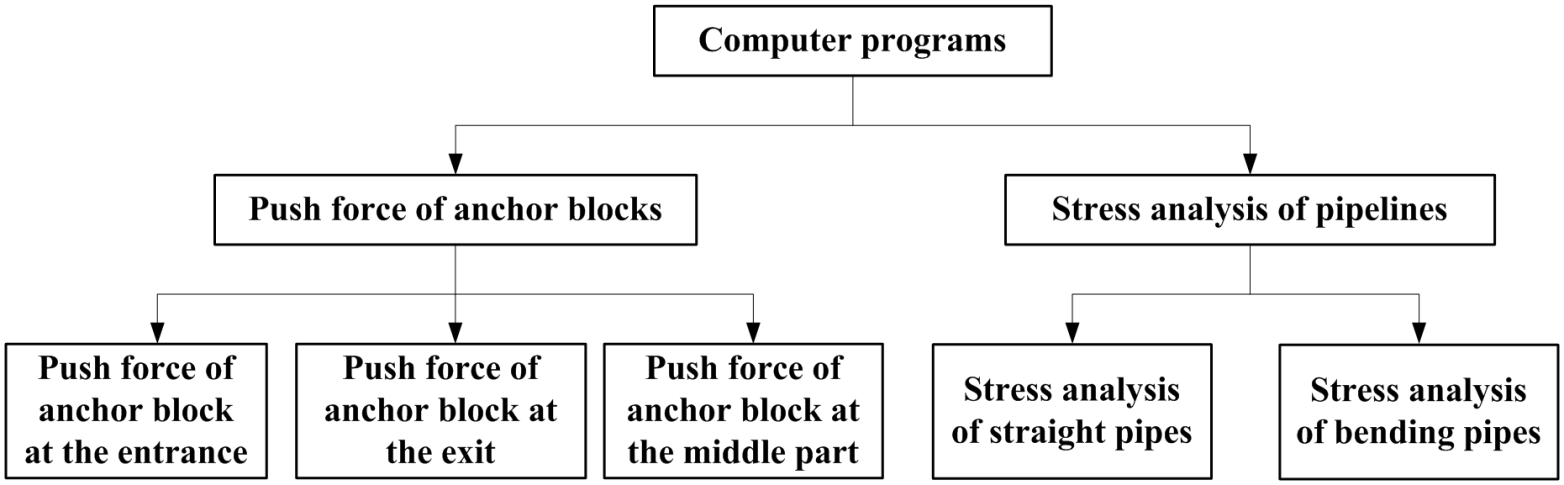
The schematic diagram of the computer program.

In China, West-East gas pipeline system project was constructed to relieve the energy market of eastern cities, having one trunk line with an outside diameter of 1219 mm. To save the engineering costs, the project crosses the complex geological areas, from Tianshan to Nanling. Given that long-distance oil and gas pipelines often pass through complex regions, different methods of crossing mountains were used in engineering. In order to reduce pipeline construction costs, mountain tunnel is commonly constructed to lay the pipeline as a reasonable and effective way. The cost of constructing gas pipelines in tunnels is usually high because the terrain on which the tunnels lie is excessively complex. Tunnel construction effectively reduces gas pipeline construction costs and links different gas tanks. To prevent damage to the tunnel or the pipeline elbow, an anchor block is set in the straight pipeline near the pipeline elbow to limit the thermal expansion displacement of the pipeline [1–3]. This displacement is caused by temperature and pressure. The key factor in anchor block design is to calculate the push force endured by this block. By searching the work published, it can be found that some researchers performed the design of the anchor block when giving a known push force [4–7]. The push force result can be solved accurately using the finite element software, but achieving this result is difficult for field staff members because of modelling and operation complexities. In this study, the virtual work principle is introduced to derive the analytic model for the anchor push force of a single-slope tunnel pipeline, induced by thermal expansion displacement. In this analysis, the statically indeterminate mechanics model of the bend was established, and the condition of deformation compatibility and the hypothesis of stagnation point were used. The corresponding computer program was developed as well (see Fig 1). The analytical calculation results are compared with the results obtained with finite element software to verify the accuracy of the formula.

## 2. Engineering model of the slope tunnel pipeline

**Fig 2:**
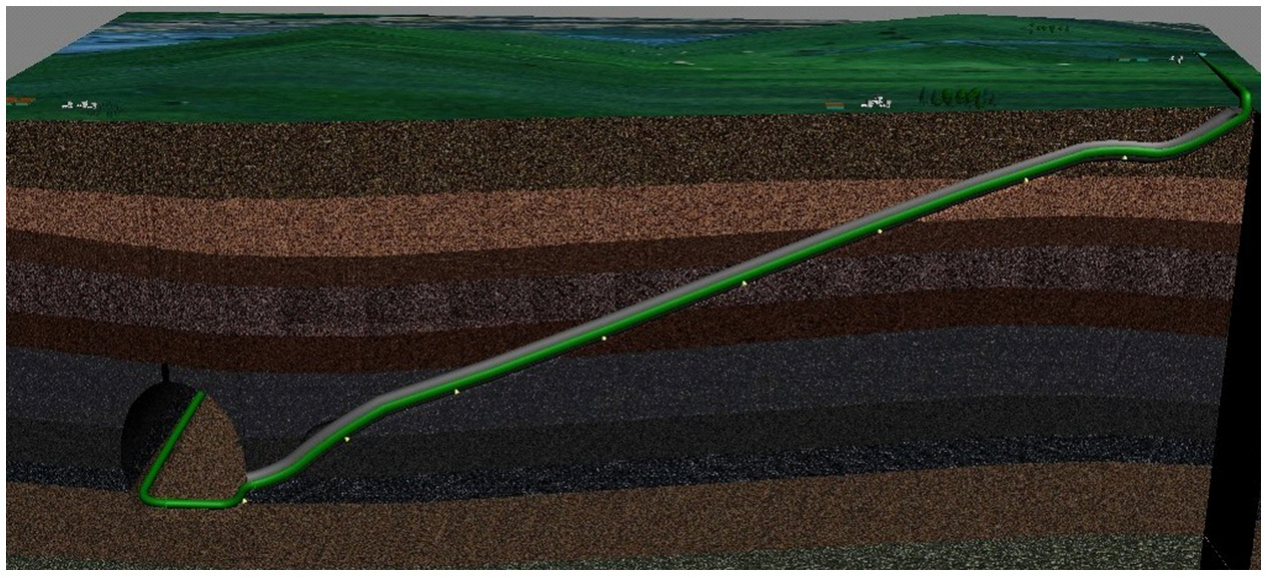
Structural representation of the single-slope tunnel pipeline in oil and gas engineering.

**Fig 3:**
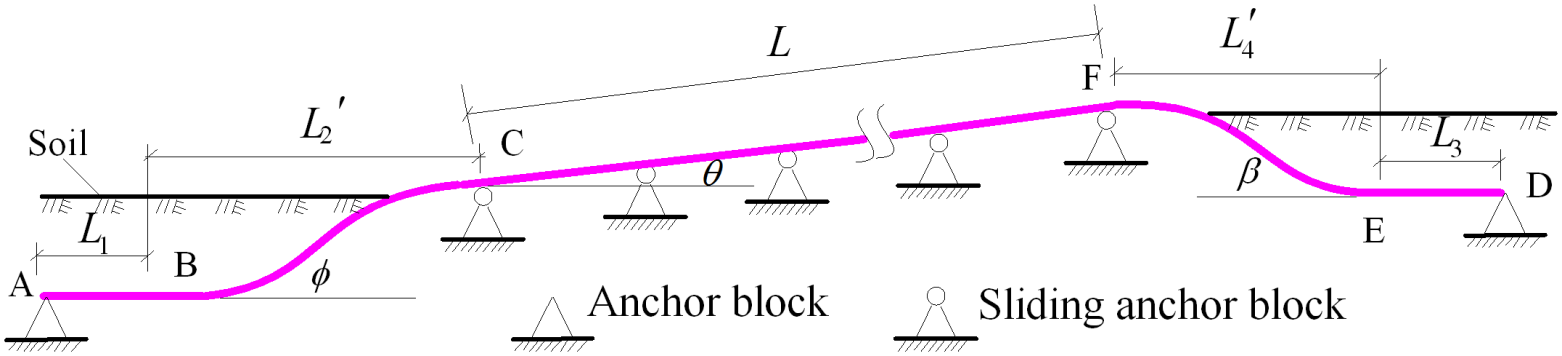
Engineering model of the slope tunnel pipeline.

The tunnel pipelines constructed for the West—East Gas Transmission Pipeline Project are laid in various forms. Amongst the slope tunnel pipelines, a vertical elbow is commonly set at the entrance and at the exit of the tunnel. The structural representation and engineering model of these pipelines are shown in Figs 2 and 3, respectively.

A tunnel pipeline with a vertical elbow is located at the tunnel opening. Two elbows are inserted into the ground in the tunnel opening, and the angles are denoted by *β* and *ϕ*. The anchor blocks are located at distances of *L*_1_ and *L*_3_ from the external elbow. The sliding anchor blocks are set continuously inside the tunnel, and the span between them is equal to *l*. The total number of span is indicated by *n*.

## 3. Mechanical simplification of the engineering model

An anchor block can prevent the pipeline from rotating and moving in any direction. The mechanical model can be modelled as the fixed-end constraint. This kind of constraint can subject the moment, axial force and shear. Meanwhile, the constraint prevents any movement.

A sliding anchor block can provide a supply at the vertical direction for the pipeline. However, this kind of the block does not provide the reaction force along the axial direction of the pipeline; nor does it supply the moment and axial force. Consequently, the pipeline can move along the axial direction. The friction force between the tunnel pipeline and the anchor can, to some extent, prevent the movement tendency of the tunnel pipeline. According to the analysis above, the mechanical model for a sliding anchor block can be simplified as an interface constraint. The accompanying friction force is exerted in the horizontal direction.

If the straight pipe is much longer than the arc-length of an elbow in the tunnel pipeline system, the internal force of the elbow section changes a little. The elbow can reduce to a hinge model, retaining the bending flexibility. This model is called the elastic bending hinge [8], regardless of the elbow size. Assuming that the moment of the elastic bending hinge is denoted by *M*, and the change in the bending resistance hinge induced by *M* is represented by Δ*φ*, then *M* and Δ*φ* should have a linear relationship. The corresponding equations are given by [9]

M=K⋅Δφ. (1)

$$K=\frac{E\pi rt^{2}}{1.65\varphi }.$$ (2)

The effect of the internal pressure can be considered as follows:

$$K=\frac{E\pi rt^{2}}{1.65\varphi }\left[1+\frac{6pr}{Et}{\left(\frac{r}{t}\right)}^{4/3}{\left(\frac{R}{r}\right)}^{1/3}\right].$$ (3)

**Fig 4:**
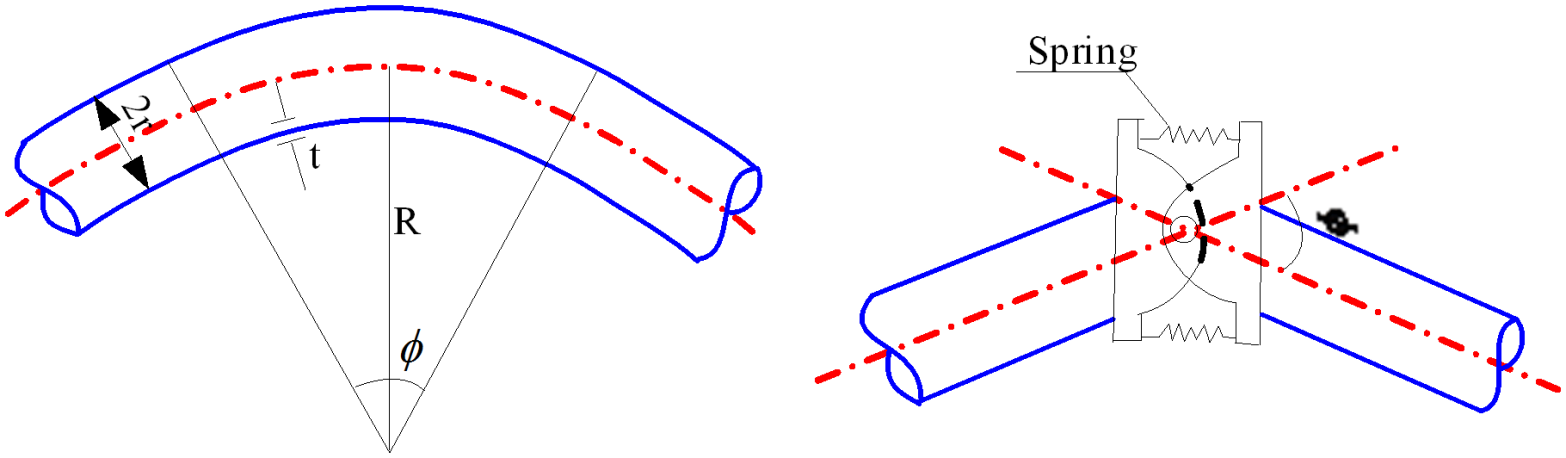
Simplified model of the elbow.

The model of the elastic bending hinge is shown in Fig 4.

**Fig 5:**
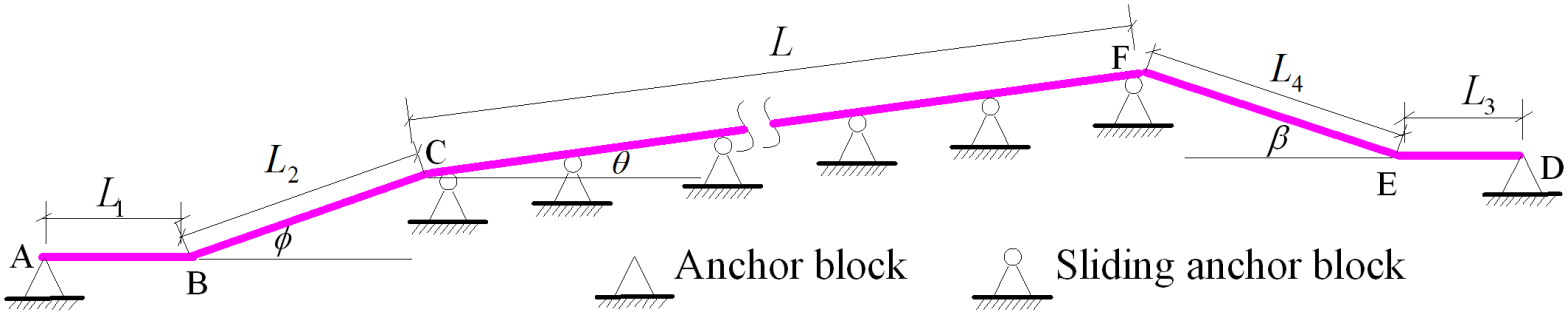
Mechanical model after simplification.

The post-simplification mechanical model is shown in Fig 5.

## 4. Stagnation point analysis

If both ends of each straight pipeline did not be constricted by the anchor block, the axial elongation induced by thermal expansion can be observed at both ends of the pipeline. Due to the different thermal expansion directions between two neighbouring straight pipes near the elbow, bending deformation was generated by push force. Note that, the bending deflection of the straight pipe can disappear rapidly due to the limitation of the anchor block. The deformation mainly occurs near the elbow [10–11].

The derivation of push force of the anchor block follows three assumptions.

1. When thermal expansion occurs, the straight pipe between two elbows elongates to the ends. A point without axial displacement certainly exists along the axial direction of the straight pipeline. The directions of friction force in the two sides between the pipe and the anchor block are opposites. Therefore, a stagnation point must be encountered between the two elbows when thermal expansion occurs.
2. Significant bending deformation can be only observed near the elbow. This deformation decreases rapidly far away from the elbow. The lateral displacement and deflection angle of the section were neglected; this point is equivalent to anchorage points.
3. The deformation in each straight pipeline is related to the nearest two elbows and straight pipelines. This deformation does not almost have the connection with the distant elbows and straight pipes. As a result, few correlations are determined amongst the positions of stationary points.

## 5. Anchor block push force based on an energy method

According to the virtual work principle [12], the deformations at both ends of a bar generated by actual loads are assumed as a virtual displacement. To solve Δ (i.e., the displacement of the section along a specified direction as a result of real loads), the unit force can be applied at this point. The internal forces of the cross-section induced by the unit force are represented by $\bar{N}$ and $\bar{M}$. The virtual work principle of the bar is given by

$$1\cdot \Delta =\int_{l}\left(\bar{N}d\delta +\bar{M}d\theta \right).$$ (4)

Eq (4) is the general expression equation of the displacement of the bar using the unit force method. The displacement generated by a real load is treated as virtual displacement [13–14], and the virtual unit force is taken as the load when the virtual displacement principle is applied. The equation, used to solve the displacement of the linear elastic body based on the unit force method, is given by

$$1\cdot \Delta \int_{0}^{l}\bar{N}\frac{Ndx}{EA}+\int_{0}^{l}\bar{M}\frac{Mdx}{EI}.$$ (5)

### 5.1. Construction of a statically indeterminate model at an elbow

**Fig 6:**
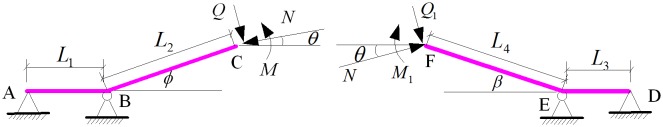
Internal force of the elbow structure at the entrance and the exit.

Considering the effect of soil reaction force [15–16] on the sections of AB and DE, the vertical displacements of points B and E are restricted. Therefore, a vertical restraint is certainly set on these points. The internal forces of the elbow in points C and F are shown in Fig 6.

**Fig 7:**
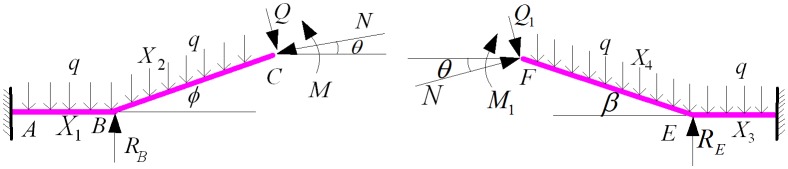
Statically indeterminate mechanical model of the elbow structure at the entrance and the exit.

Removing the constraints of point B, a statically indeterminate mechanical model of the elbow at the entrance and the exit is established. This model is presented in Fig 7.

Based on the fact that the deflection values of B and E were equal to 0, *R*_*B*_ and *R*_*E*_ in the indeterminate structure are obtained.

$$R_{B}=-\frac{3\left[M+NL_{2}\text{sin}\left(\varphi -\theta \right)-QL_{2}\text{sin}\left(\varphi -\theta \right)\right]}{2L_{1}}+Q.$$ (6)

$$R_{E}=-\frac{3\left[M_{1}+NL_{4}\text{sin}\left(\beta +\theta \right)-Q_{1}L_{4}\text{sin}\left(\beta +\theta \right)\right]}{2L_{3}}+Q_{1}.$$ (7)

### 5.2. Research on the joint displacements of points C and F at an elbow using an energy method

**Fig 8:**
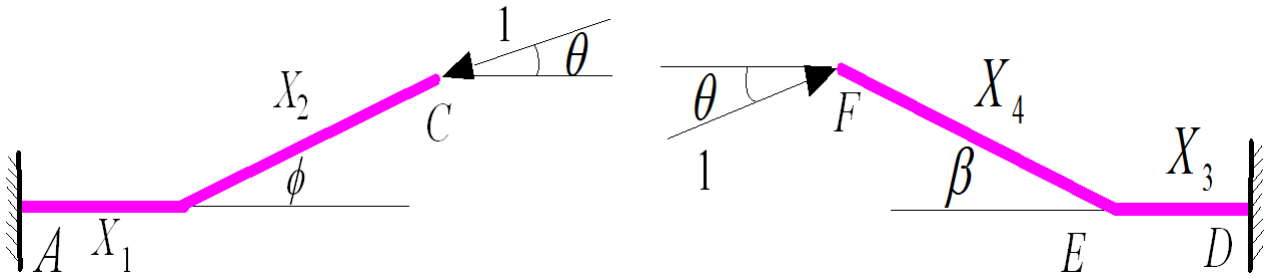
Elbow structure established by applying unit force to the entrance and the exit.

The force analysis generated by applying the unit force in points C and F are displayed in Fig 8.

The displacements of points C and F are defined as *f*_*c*_ and *f*_*F*_, respectively. These variables can be expressed as the following formula based on the unit force method. Additionally, the displacements of points C and F are along the axial direction.

$$f_{c}=N\left[\frac{L_{1}{\text{cos}}^{2}\theta +L_{2}{\text{cos}}^{2}\left(\varphi -\theta \right)}{EA}+\frac{\left(4L_{2}^{3}+3L_{1}L_{2}^{2}\right){\text{sin}}^{2}\left(\varphi -\theta \right)\text{sin}\theta +4\text{sin}\theta^{2}L_{1}^{3}}{12EI}\right]+\Delta_{1}+\Delta_{2}$$

$$f_{F}=N\left[\frac{L_{3}+L_{4}{\text{cos}}^{2}\beta }{EA}+\frac{3L_{3}L_{4}^{2}{\text{sin}}^{2}\beta +4L_{4}^{3}{\text{sin}}^{2}\beta }{12EI}\right]+\Delta_{3}+\Delta_{4},$$

where

$$\Delta_{1}=\frac{1}{EA}\left[QL_{2}\text{sin}\left(\varphi -\theta \right)\text{cos}\left(\varphi -\theta \right)+q\frac{L_{2}^{2}}{2}\text{sin}\varphi \text{cos}\left(\varphi -\theta \right)\right]$$

$$\Delta_{2}=\frac{1}{EI}\{ML_{2}\text{sin}\left(\varphi -\theta \right)\left(\frac{L_{1}}{4}+\frac{L_{2}}{2}\right)-Q[L_{2}^{2}\left(\frac{L_{1}}{8}+\frac{L_{2}}{6}\right)\text{sin}\left(2\varphi -2\theta \right)+\frac{L_{1}^{2}L_{2}}{2}\text{cos}\theta \text{sin}\left(\varphi -\theta \right)-\frac{L_{1}^{2}L_{2}}{2}\text{sin}\left(\varphi -\theta \right)-\frac{L_{1}^{3}}{6}\text{sin}2\theta +\frac{L_{1}^{3}}{3}\text{sin}\theta ]-\frac{qL_{1}^{3}L_{2}}{6}\text{sin}\left(\varphi -\theta \right)+\frac{qL_{1}^{4}}{8}\text{sin}\theta \}$$

$$\Delta_{3}=\frac{1}{EA}\left(Q_{1}L_{4}\text{sin}\beta \text{cos}\beta +q\frac{L_{4}^{2}}{2}\text{sin}\beta \text{cos}\beta \right)$$

$$\Delta_{4}=\frac{L_{4}\text{sin}\beta }{EI}\left[\left(\frac{L_{4}}{2}+\frac{L_{3}}{4}\right)M_{1}-\left(\frac{L_{4}^{2}\text{cos}\beta }{3}+\frac{1}{4}L_{3}L_{4}\text{cos}\beta \right)Q_{1}-\frac{qL_{3}^{3}}{6}\right]$$

The straight pipeline between two elbows extends to each other, when the pipeline is in expansion case. A point without any axial displacement certainly exists along the axial direction. The direction of support friction on either end of the point is opposite that of soil friction. This point is called the stagnation point. Assuming that the distance between the stagnation point of and point C is *L*_5_, and the distance between this point and point F is *L*_6_. Under pressure *P* and axial force *N*, the displacement of *μ*_*c*_ can be determined as follow:

$$u_{c}=\alpha \Delta t\left(L_{1}\text{cos}\theta +L_{2}\text{cos}\left(\phi -\theta \right)+L_{5}\right)-\frac{N}{EA}\left(L_{1}\text{cos}\theta +L_{2}\text{cos}\left(\phi -\theta \right)+L_{5}\right)+\left(1-2\nu \right)\frac{\pi r^{2}P}{EA}\left(L_{1}\text{cos}\theta +L_{2}\text{cos}\left(\phi -\theta \right)+L_{5}\right).$$

At point C, the displacement also satisfies the deformation compatibility condition, i.e., *f*_*c*_ = *μ*_*c*_. Thus,

$$N=\frac{\left(\alpha \Delta t+\left(1-2\nu \right)\frac{\pi r^{2}P}{EA}\right)\left(L_{1}\text{cos}\theta +L_{2}\text{cos}\left(\varphi -\theta \right)+L_{5}\right)-\Delta_{1}-\Delta_{2}}{\Delta_{5}}.$$ (8)

Similarly, using the deformation compatibility condition at point F, i.e., *f*_*F*_ = *μ*_*F*_, the corresponding equation can be expressed as follow:

$$N=\frac{\left(\alpha \Delta t+\left(1-2\nu \right)\frac{\pi r^{2}P}{EA}\right)\left(L_{3}\text{cos}\theta +L_{4}\text{cos}\left(\beta +\theta \right)+L_{6}\right)-\Delta_{3}-\Delta_{4}}{\Delta_{6}}.$$ (9)

Where:

$$\Delta_{5}=\frac{L_{1}{\text{cos}}^{2}\theta +L_{2}{\text{cos}}^{2}\left(\varphi -\theta \right)}{EA}+\frac{\left(4L_{2}^{3}+3L_{1}L_{2}^{2}\right){\text{sin}}^{2}\left(\varphi -\theta \right)-6L_{1}^{2}L_{2}\text{sin}\left(\varphi -\theta \right)\text{sin}\theta +4\text{sin}\theta^{2}L_{1}^{3}}{12EI}+\frac{L_{1}\text{cos}\theta +L_{2}\text{cos}(\phi -\theta )+L_{5}}{EA}$$

$$\Delta_{6}=\frac{L_{3}{\text{cos}}^{2}\theta +L_{4}{\text{cos}}^{2}\left(\beta +\theta \right)}{EA}+\frac{\left(4L_{4}^{3}+3L_{3}L_{4}^{2}\right){\text{sin}}^{2}\left(\beta +\theta \right)+6L_{3}^{2}L_{4}\text{sin}\left(\beta +\theta \right)\text{sin}\theta +4\text{sin}\theta^{2}L_{3}^{3}}{12EI}+\frac{L_{3}\text{cos}\theta +L_{4}\text{cos}\left(\beta +\theta \right)+L_{6}}{EA}$$

Summarising the two equations above, it can be obtained as follow:

$$\frac{\left(\alpha \Delta t+\left(1-2\nu \right)\frac{\pi r^{2}P}{EA}\right)\left(L_{1}\text{cos}\theta +L_{2}\text{cos}\left(\varphi -\theta \right)+L_{5}\right)-\Delta_{1}-\Delta_{2}}{\Delta_{5}}=\frac{\left(\alpha \Delta t+\left(1-2\nu \right)\frac{\pi r^{2}P}{EA}\right)\left(L_{3}\text{cos}\theta +L_{4}\text{cos}\left(\beta +\theta \right)+L_{6}\right)-\Delta_{5}-\Delta_{6}}{\Delta_{6}}.$$ (10)

When the soil breakout friction in sections *L*_1_ and *L*_3_ are neglected, *N* is the thrust of the anchor block.

### 5.3. Approximate solution of moment and shear

Assuming that no elbow exists in the middle of the slope tunnel pipeline, only one stagnation point exists, and

$$L_{5}+L_{6}=L.$$ (11)

Assuming that the friction forces of the sliding anchor block were loaded uniformly at the pipeline, the deflection equation can be solved according to the one of a buried vertical pipe. Then, the deflection equation of *ω* can be expressed as [17]

$$\omega ≅\left\{ \begin{array}{l} \left(\alpha \Delta tL-\frac{\nu qL^{2}}{2EA}\right)\left(1-\frac{x}{l_{b}}\right)\left(1+\frac{5x}{2l_{b}}\right)\;\left(0\leq x\leq l_{b}\right)\\ 0 \end{array}\right.,$$

where *l*_*b*_ is the approximate location of the first zero point.

$$l_{b}=\sqrt[{4}]{\frac{30\sqrt{2}EF\alpha \Delta tLr^{2}}{\mu q}}.$$

**Fig 9:**
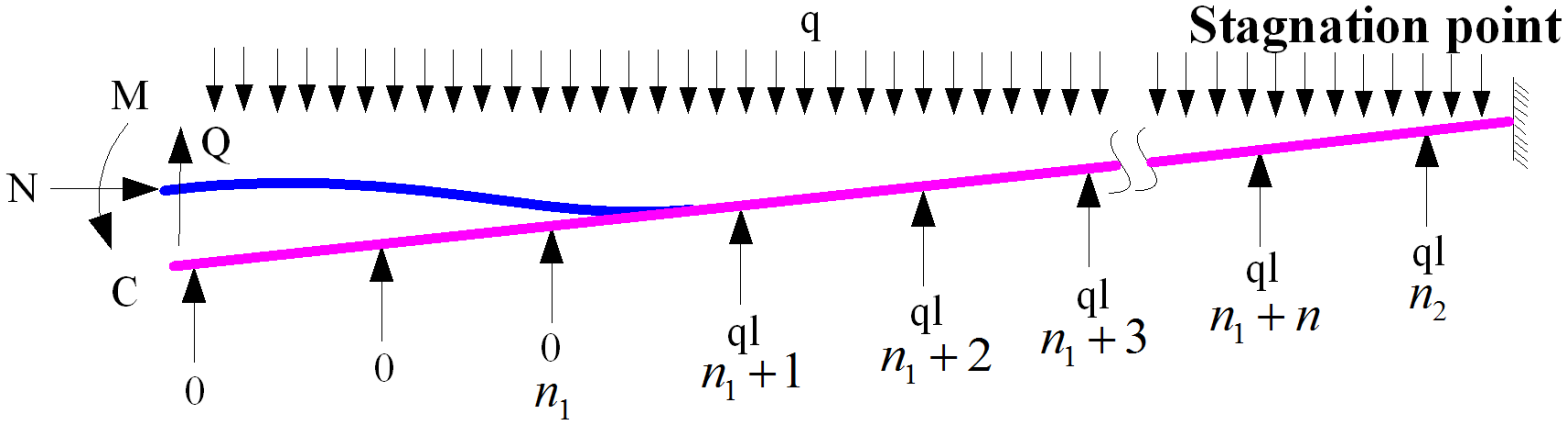
Calculation of M and Q values after pipeline flexure deformation.

The model, used to solve the values of *M* and *Q*, is shown in Fig 9.

*l*_*a*_ is the distance between the deflection point with the values of 0 and the elbow (point C) in the portal and is given by (Zhang et al., 2015)

$$l_{a}=\sqrt[{4}]{\frac{30\sqrt{2}EF\alpha \Delta tLr^{2}}{\mu q}}=\sqrt[{4}]{\frac{30\sqrt{2}EF\alpha \Delta tL_{5}r^{2}}{\mu q}},$$

where *L*_5_ is the distance from the stagnation point to point C.

The following assumption is considered in this analysis. The vertical reaction force in the middle anchor block is calculated with *F*_*Z*_ = *ql*, and *l* is the span between two sliding anchor blocks. Therefore, the shearing force of point C is written as

$$Q=ql_{a}\text{cos}\theta .$$ (12)

Assuming that *n*_1_ is the integer value of *l*_*a*_/*l* and *n*_2_ is the integer value of *l*_5_/*l*, the moment of *M* at the point C can be expressed as

$$M=q\frac{L_{5}^{2}\text{cos}\theta }{2}-ql^{2}\frac{\left(n_{2}-n_{1}\right)\left(n_{1}+1+n_{2}\right)}{2}+\frac{ql^{2}}{12}.$$ (13)

*l*_*b*_ is the distance between the deflection point with the values of 0 and elbow (point F) in the opening and is given by

$$l_{b}=\sqrt[{4}]{\frac{30\sqrt{2}EF\alpha \Delta tLr^{2}}{\mu q}}=\sqrt[{4}]{\frac{30\sqrt{2}EF\alpha \Delta tL_{6}r^{2}}{\mu q}},$$

where *L*_6_ is the distance from the stagnation point to point F.

Based on assumption above, the shearing force *Q*_1_ at the point F is written as

$$Q_{1}=ql_{b}\text{cos}\theta .$$ (14)

If *n*_3_ is the integer value of *l*_*b*_/*l* and *n*_4_ is the integer value of *l*_*6*_/*l*, then the moment *M*_1_ of point F can be expressed as

$$M_{1}=q\frac{L_{6}^{2}\text{cos}\theta }{2}-ql^{2}\text{cos}\theta \frac{\left(n_{4}-n_{3}\right)\left(n_{3}+1+n_{4}\right)}{2}+\frac{ql^{2}}{12}.$$ (15)

According to Eqs (10)–(15), six unknown variables (*M*, *Q*, *M*_1_, *Q*_1_, *L*_5_ and *L*_6_) can be solved. The thrust of *N* can be solved by substituting the results into Eqs (7) or (8).

### 5.4. Analytical formula of anchor block push force

In the case of thermal expansion, the straight pipe between two elbows elongates to each other. A point without axial displacement certainly exists along the axial direction. The directions of friction force oppose each other at its two sides. As a result, a stagnation point must be detected between the two elbows when thermal expansion occurs.

The displacement at point C under the effects of temperature difference Δ*t*, pressure *P* and axial force *N* is indicated by *μ*_*c*_.

$$u_{c}=\left[\alpha \Delta t+\left(1-2\nu \right)\frac{\pi r^{2}P}{EA}\right]\left[\left(L_{1}\text{cos}\theta +L_{2}\text{cos}\left(\varphi -\theta \right)+L_{5}\right)\right]-\frac{N}{EA}\left[\left(L_{1}\text{cos}\theta +L_{2}\text{cos}\left(\varphi -\theta \right)+L_{5}\right)\right]$$

Considering the friction of the pipe pier, the friction is unrelated to bending moments *M* and *M*_1_ when the friction direction passes through points C and F. Eqs (8) and (9) are then written as

$$N=\frac{\left[\alpha \Delta t+\left(1-2\nu \right)\frac{\pi r^{2}P}{EA}\right]\left[L_{1}\text{cos}\theta +L_{2}\text{cos}\left(\varphi -\theta \right)+L_{5}\right]-\Delta_{1}-\Delta_{2}-\frac{\mu qL_{5}^{2}}{2EA}}{\Delta_{5}}$$ (16)

$$N^{\prime }=\frac{\left[\alpha \Delta t+\left(1-2\nu \right)\frac{\pi r^{2}P}{EA}\right]\left[L_{3}\text{cos}\theta +L_{4}\text{cos}\left(\beta +\theta \right)+L_{6}\right]-\Delta_{3}-\Delta_{4}-\frac{\mu qL_{6}^{2}}{2EA}}{\Delta_{6}}$$ (17)

According to the equilibrium equation at the horizontal direction, *N−μqL*_5_ = *N′−μqL*_6_, Eqs (16) and (17) can be further expressed as:

$$\frac{\left[\alpha \Delta t+\left(1-2\nu \right)\frac{\pi r^{2}P}{EA}\right]\left[L_{1}\text{cos}\theta +L_{2}\text{cos}\left(\varphi -\theta \right)+L_{5}\right]-\Delta_{1}-\Delta_{2}-\frac{\mu qL_{5}^{2}}{2EA}}{\Delta_{5}}-\mu qL_{5}=\frac{\left[\alpha \Delta t+\left(1-2\nu \right)\frac{\pi r^{2}P}{EA}\right]\left[L_{3}\text{cos}\theta +L_{4}\text{cos}\left(\beta +\theta \right)+L_{6}\right]-\Delta_{5}-\Delta_{6}-\frac{\mu qL_{6}^{2}}{2EA}}{\Delta_{6}}-\mu qL_{6}.$$ (18)

Eq (18) can be used to calculate the push force of the anchor block when considering the friction effect of the pipe, instead of Eq (10).

Note that, the stress level is very important to evaluate the safety of the pipeline. Considering the relation of the stress and internal force (i.e., the axial force and bending moment), the axial stress can be solved as follow:

$$\sigma =\frac{N}{A}+\frac{MD}{2I}$$

where *N* is the axial force of the pipeline and can be solved based on Eq (17), *A* is the area of the cross-section, *M* is the bending moment and can be calculated based on Eqs (13) and (15), *D* is the external diameter, *I* is the moment of inertia.

## 6. Validation of analytical method

**Fig 10:**
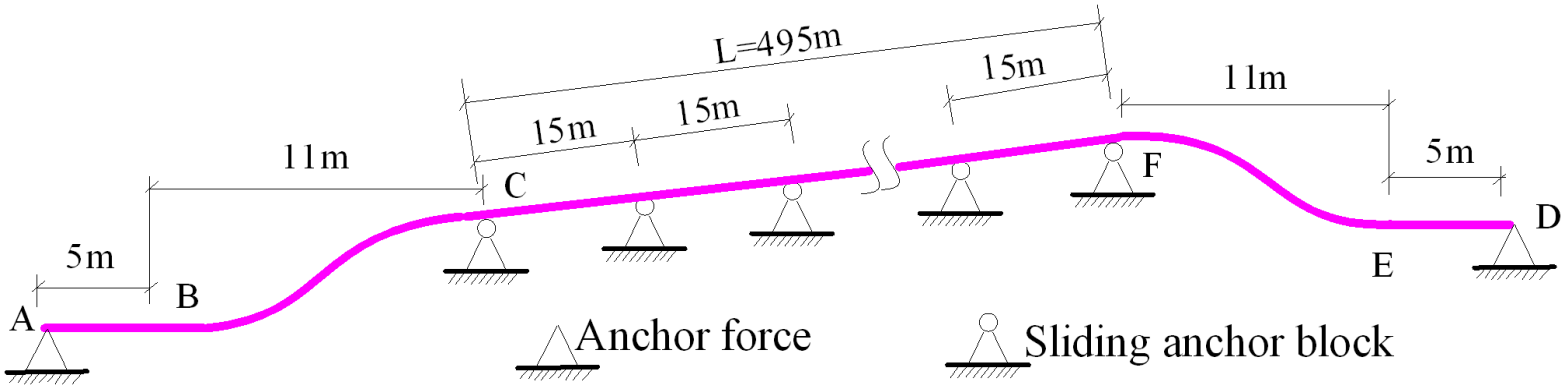
Integral engineering model of the slope tunnel pipeline.

**Fig 11:**
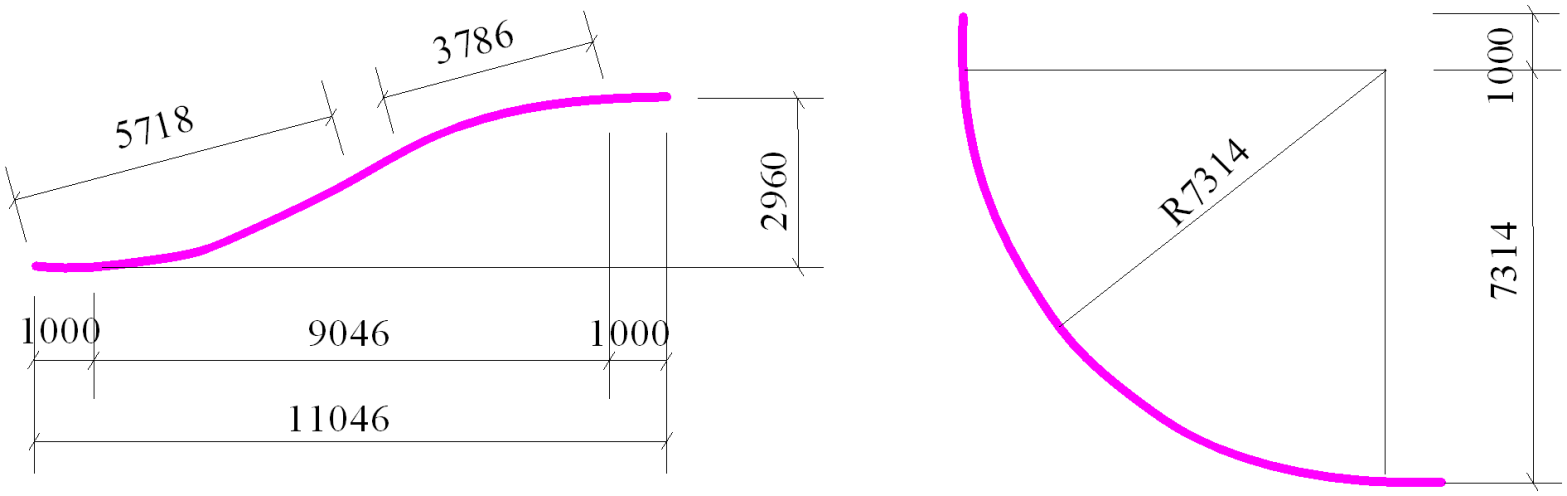
Local engineering model in and out of the opening.

To verify the accuracy of the analytical method, the results of the analytical and the finite element method for the push force of the anchor block of the single-slope tunnel pipeline from the West—East Project are compared. The pipeline is linked to an underground gas storage area containing naturally bedded rock salt. The constructional and integral engineering models of the slope tunnel pipeline are shown in Fig 10. Fig 11 depicts the engineering model in and out of the tunnel opening.

Fig 10 shows the basic parameters of the slope tunnel pipeline. The length of the slope tunnel pipeline is 495 m. Two elbows are inserted into the ground; each end is inserted with an angle of 30°. The anchor block is located at an area where the distance is 5 m from the external elbow. The elbow combination is shown in Fig 11.

The pipeline material is API 5L X80, with the yield and tensile strengths of 485 and 570 MPa, the pipeline diameter of 1219 mm, the wall thicknesses of 18.4, 22 and 26.4 mm, the design pressure of 12 MPa, the temperature difference of 40°C, the friction factor between the pipeline and the sliding anchor block of 0.6 and the weight per unit length (including the insulation layer, corrosion proofing layer and gas) of 1000 N/m.

Considering the symmetry of the tunnel pipeline structure, the stagnation point is positioned in the middle of the horizontal segment. When the pipeline diameter is equal to 1219 mm, the wall thickness is 18.4 mm. Eqs (12) and (13) indicate that *Q* = 438202.59 N and *M* = 14687071.875 N.m. The thrust *N* = 4700801.09 N is deduced using Eq (16). Tables 1 and 2 list the results of the push force of the anchor block at the entrance and exit under different tunnel gradient varies.

**Table 1:** Results for push force of anchor block at the tunnel entrance when the tunnel gradient varies.

Pipeline type	Result for anchor block push force / kN					
	5°	10°	15°	20°	25°	30°
X80, ϕ1016 × 14.6	1701	1558	1409	1202	1013	813
X80, ϕ1016 × 17.5	2189	2018	1842	1618	1381	1144
X80, ϕ1016 × 21.0	2764	2564	2357	2046	1821	1538
X80, ϕ1016 × 26.2	3649	3426	3112	2795	2416	2117

**Table 2:** Results for the anchor block push force at the tunnel exit when the tunnel gradient varies.

Pipeline type	Result for anchor block push force / kN					
	5°	10°	15°	20°	25°	30°
X80, ϕ1016 × 14.6	1762	1634	1512	1356	1231	1112
X80, ϕ1016 × 17.5	2275	2113	1976	1782	1634	1475
X80, ϕ1016 × 21.0	2869	2691	2512	2235	2093	1886
X80, ϕ1016 × 26.2	3765	3574	3297	2998	2703	2496

To verify the accuracy of the analytical method, four finite element software programs (CAESARII, ANSYS, AutoPIPE and ALGOR) were used. According to the engineering model, boundary conditions were concluded as follow: (1) the fixed constraints were used in the locations A and D, respectively. (2) the roller constraints were used in central part of the pipeline. Additionally, the loads were applied based on design parameters, including the inner pressure of 12 MPa and the temperature variation of 40°C.

Tables 3 and 4 depict comparisons of the analytical results with the numerical results calculated with different software programmes.

**Table 3:** Comparison of anchor block push forces under the operating condition at the tunnel entrance.

Pipeline type /mm	Result for anchor block push force / kN				Analytical results	Relative error *δ*
	AutoPIPE	CAESARII	ALGOR	ANSYS		
ϕ1219 × 18.4	2081	1952	1982	2125	2034	3.4%
φ1219 × 22.0	2253	2091	2124	2284	2193	3.7%
φ1219 × 26.4	2412	2225	2257	2433	2326	4.0%

**Table 4:** Comparison of anchor block push force under the operating condition at the tunnel exit.

Pipeline type	Result for anchor block push force / kN					Relative error *δ*
	AutoPIPE	CAESARII	ALGOR	ANSYS	Analytical results	
ϕ1219 × 18.4	2371	2221	2253	2413	2314	3.4%
φ1219 × 22.0	2543	2392	2431	2581	2491	3.1%
φ1219 × 26.4	2742	2534	2572	2782	2662	4.1%

*δ* is the relative error of the analytical and numerical results. The results obtained from AutoPIPE, CAESARII, ALGOR, ANSYS and the analytical formula are equal to *S*_1_, *S*_2_, *S*_3_, *S*_4_ and *S*, respectively. Then, this error can be expressed as:

$$\delta =\sqrt{\frac{{\left(S_{1}-S\right)}^{2}+{\left(S_{2}-S\right)}^{2}+{\left(S_{3}-S\right)}^{2}+{\left(S_{4}-S\right)}^{2}}{4}}/\frac{\left(S_{1}+S_{2}+S_{3}+S_{4}\right)}{4}.$$

The push force of the anchor block increases with an increase of pipe diameter and thickness. The relation between these variables is approximately linear. Considering that the tunnel pipeline is not symmetric in this example, the stagnation point is not located at the middle of the slope tunnel, positioning 220 m from point C. The stagnation point between 220/495 and the angle of two elbows (24/60) is approximately equal.

**Fig 12:**
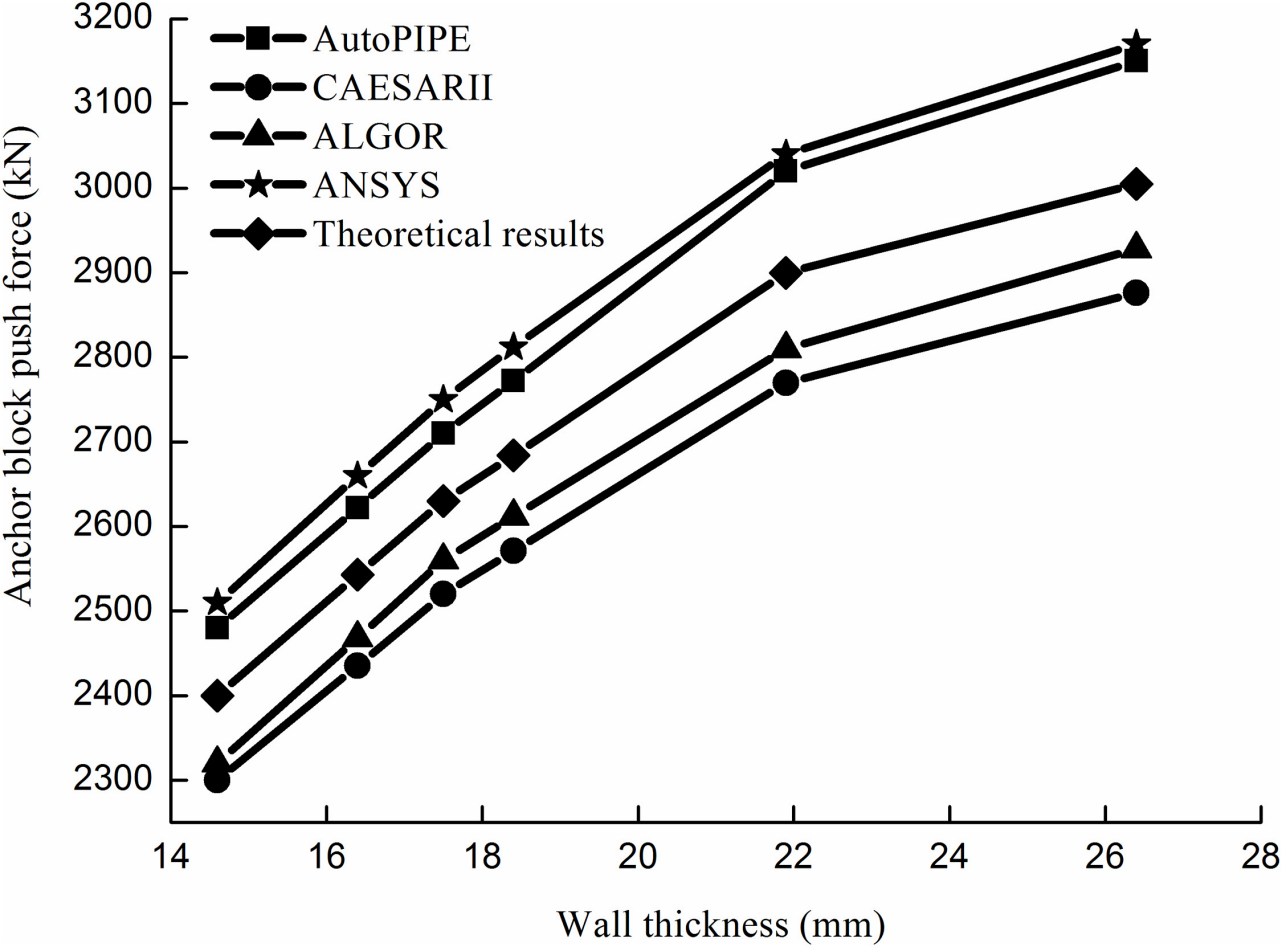
Block push force results with a pipeline diameter of 1016 mm.

**Fig 13:**
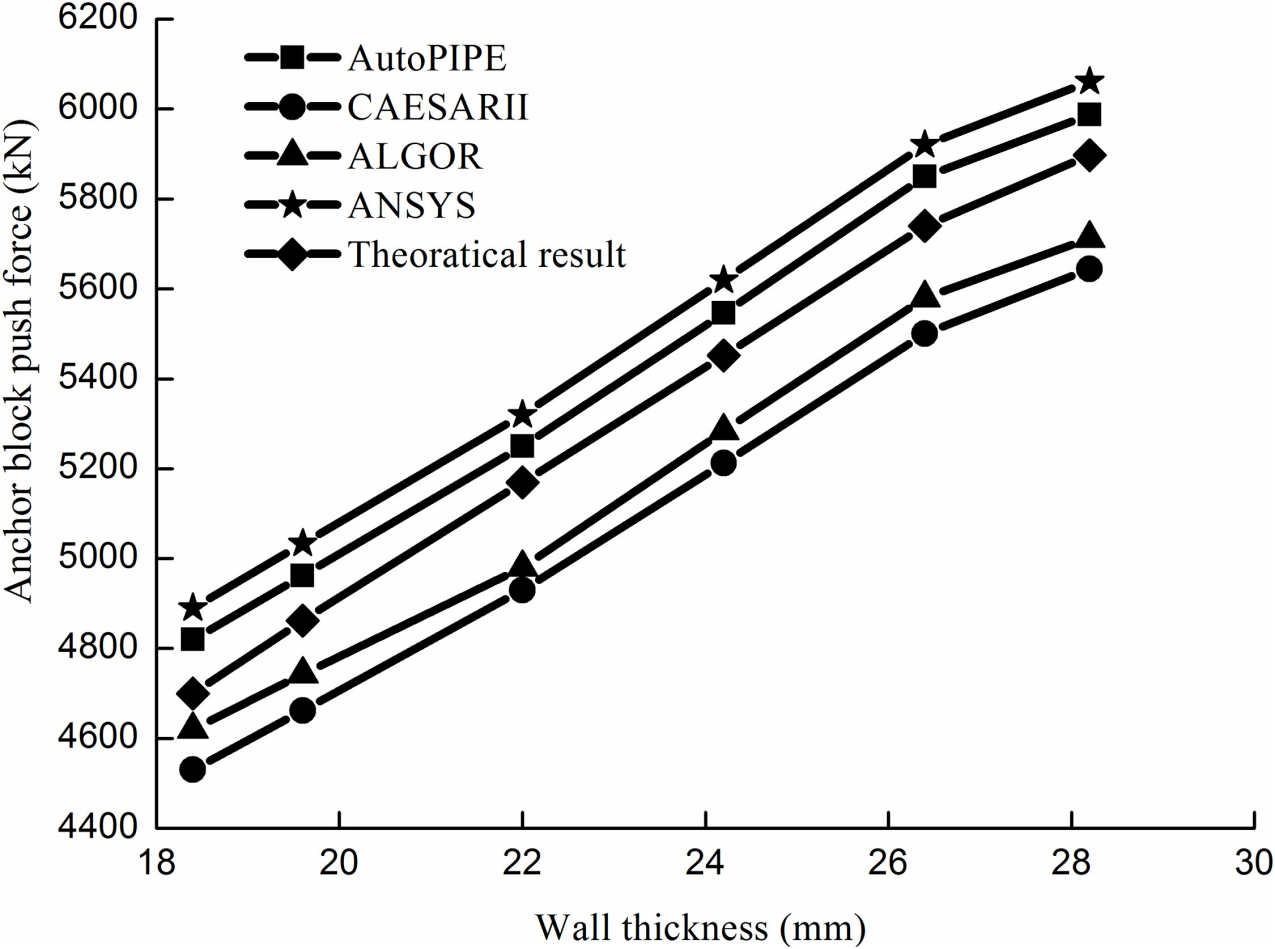
Block push force results with a pipeline diameter of 1219 mm.

Figs 12 and 13 shows the comparisons of the results between the analytical method and the numerical method for the push force of the anchor block.

The results show that the analytical and numerical results agree well each other and that the maximum relative error does not exceed 4.1%. Therefore, the results of the analytical method can satisfy engineering requirements.

Additionally, the scene pictures from the West—East Gas Project, China, were shown in Figs 14–21.

**Fig 14:**
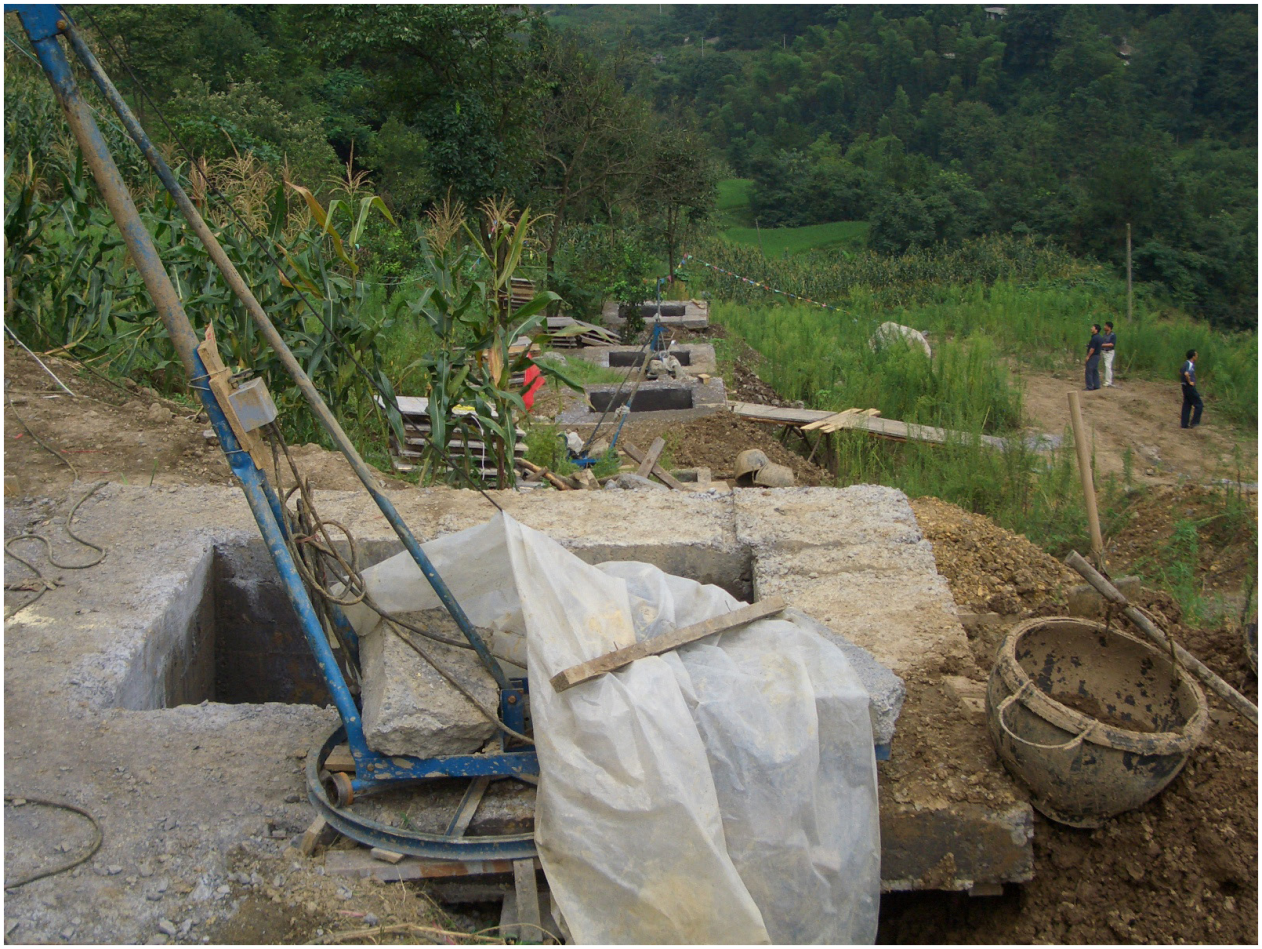
Anchor blocks at entrances.

**Fig 15:**
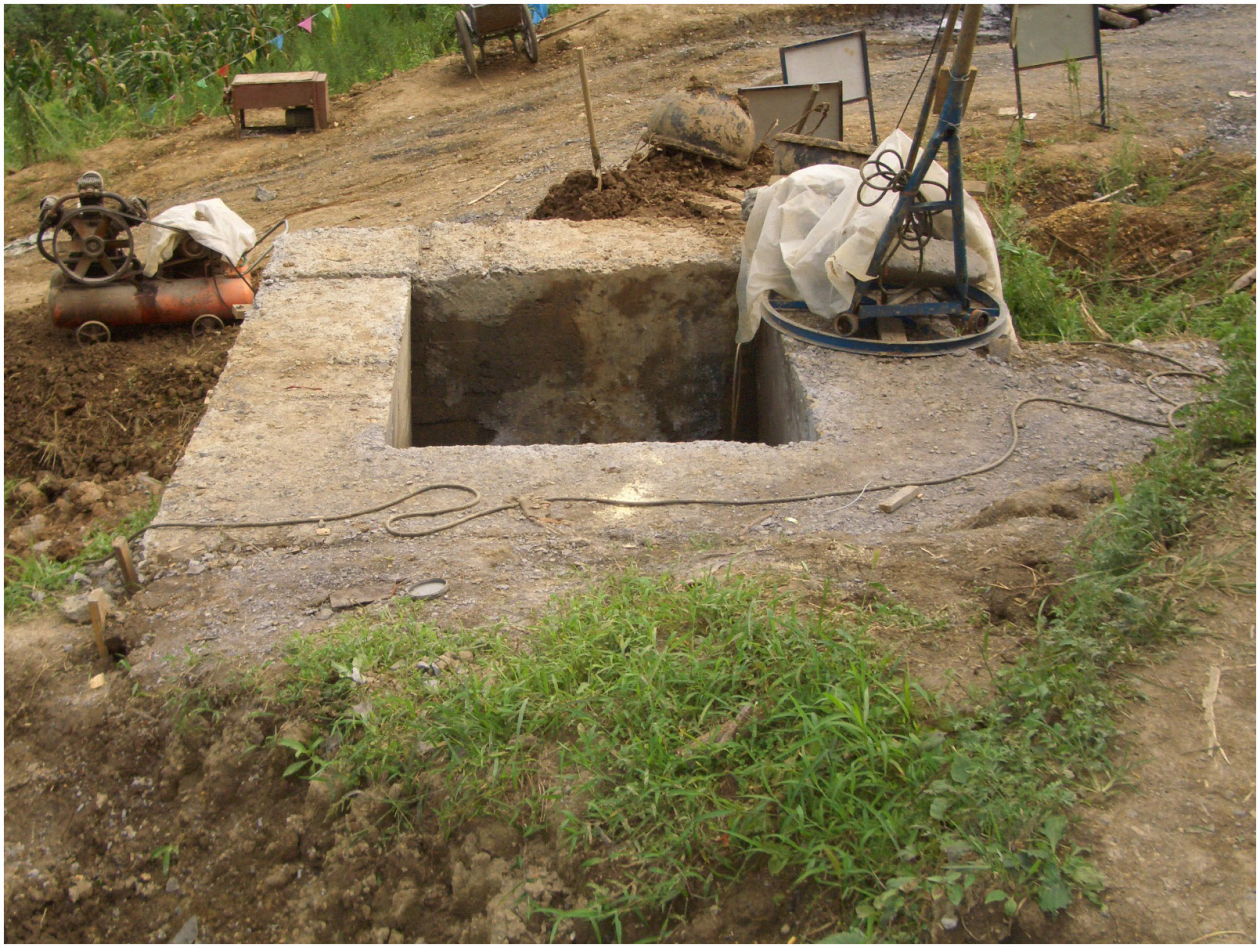
Anchor blocks at exits.

**Fig 16:**
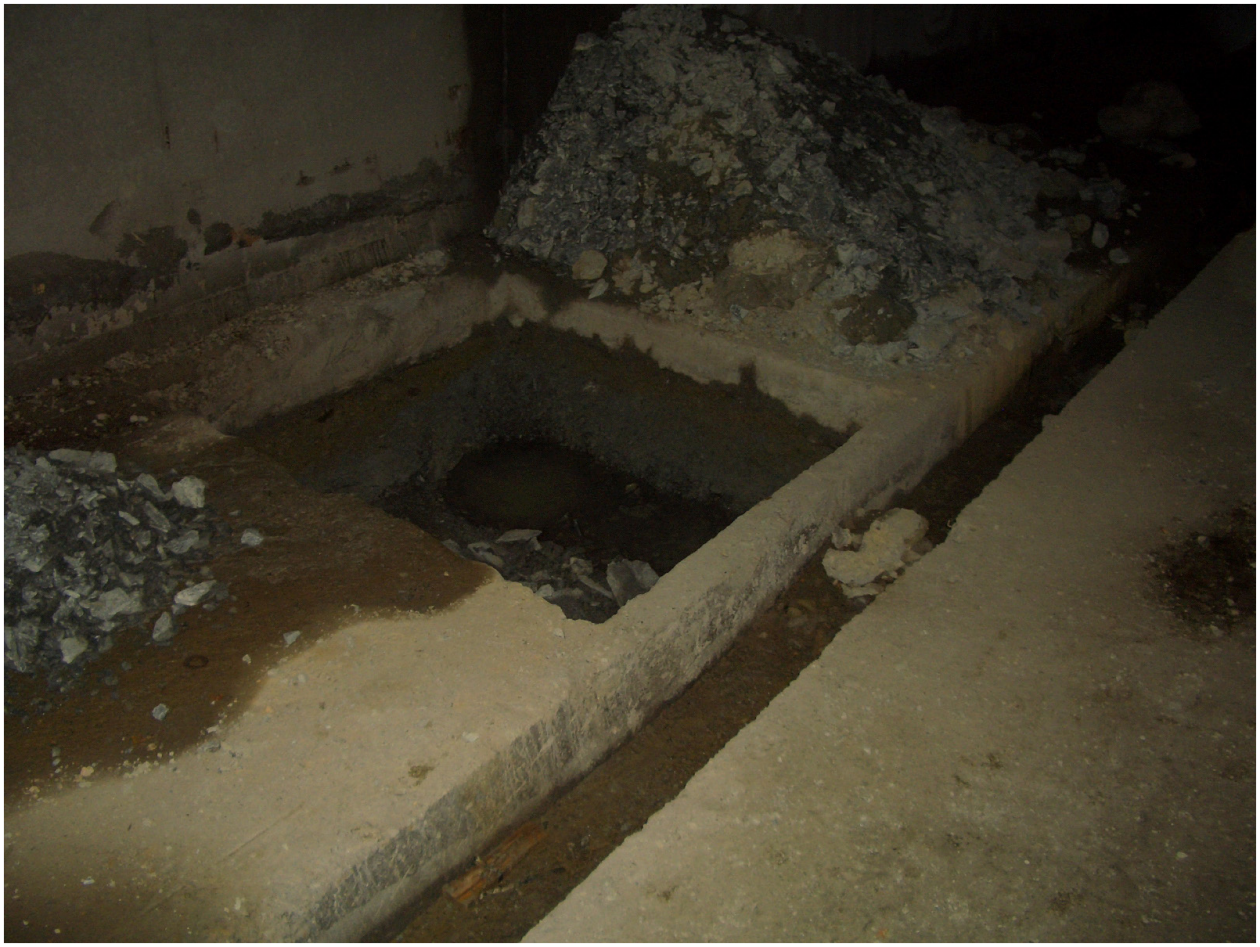
Concrete sliding blocks.

**Fig 17:**
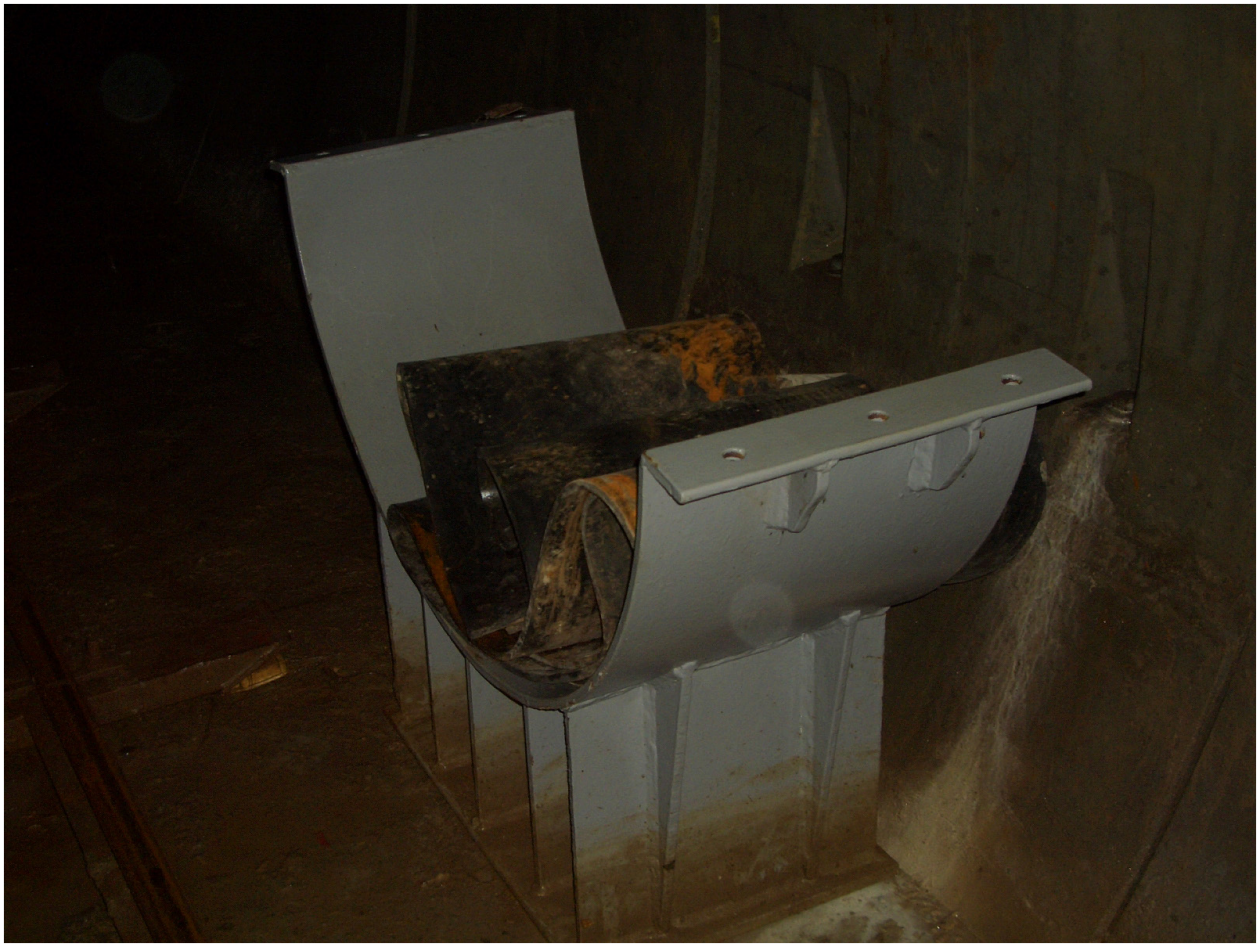
Steel sliding blocks.

**Fig 18:**
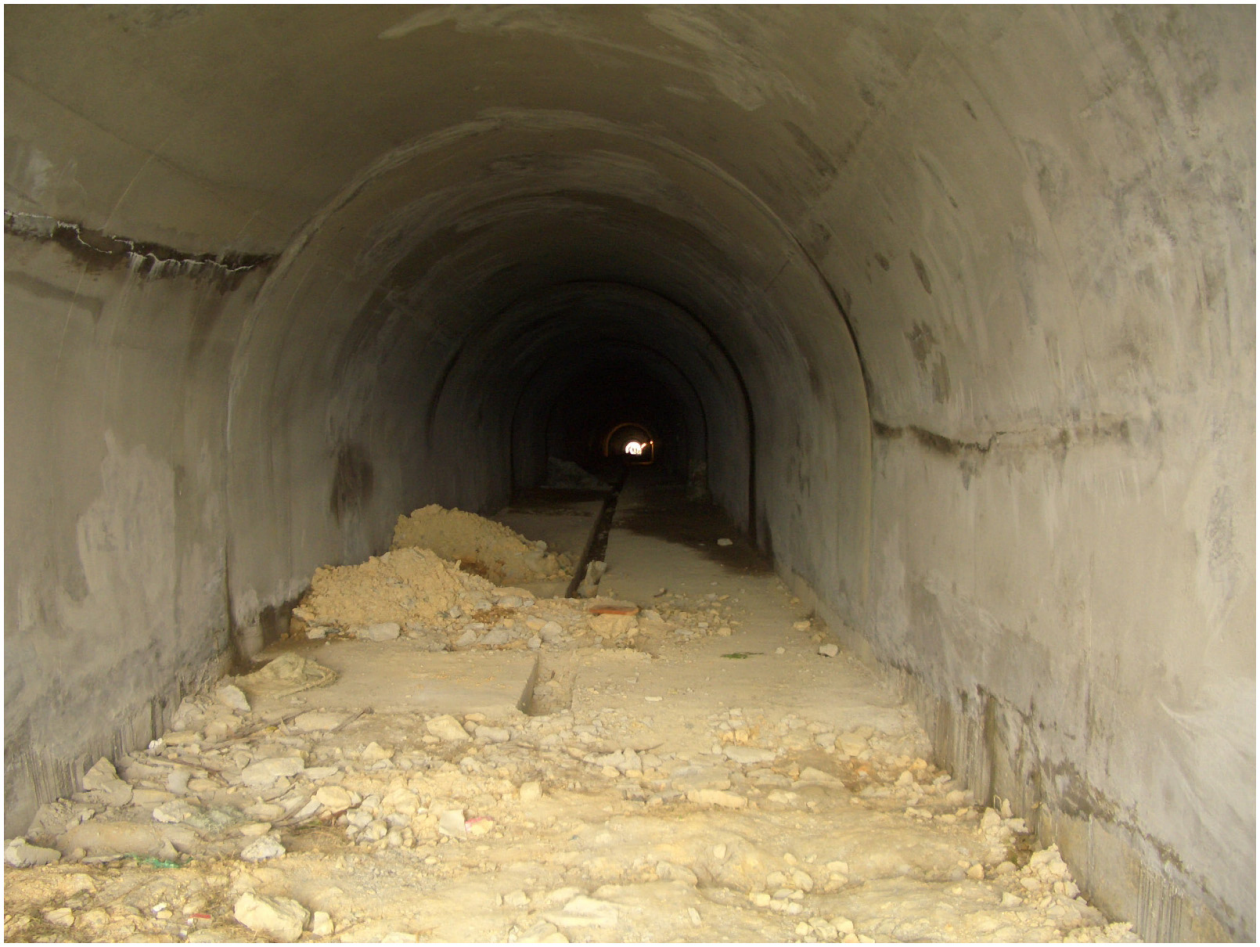
Tunnels.

**Fig 19:**
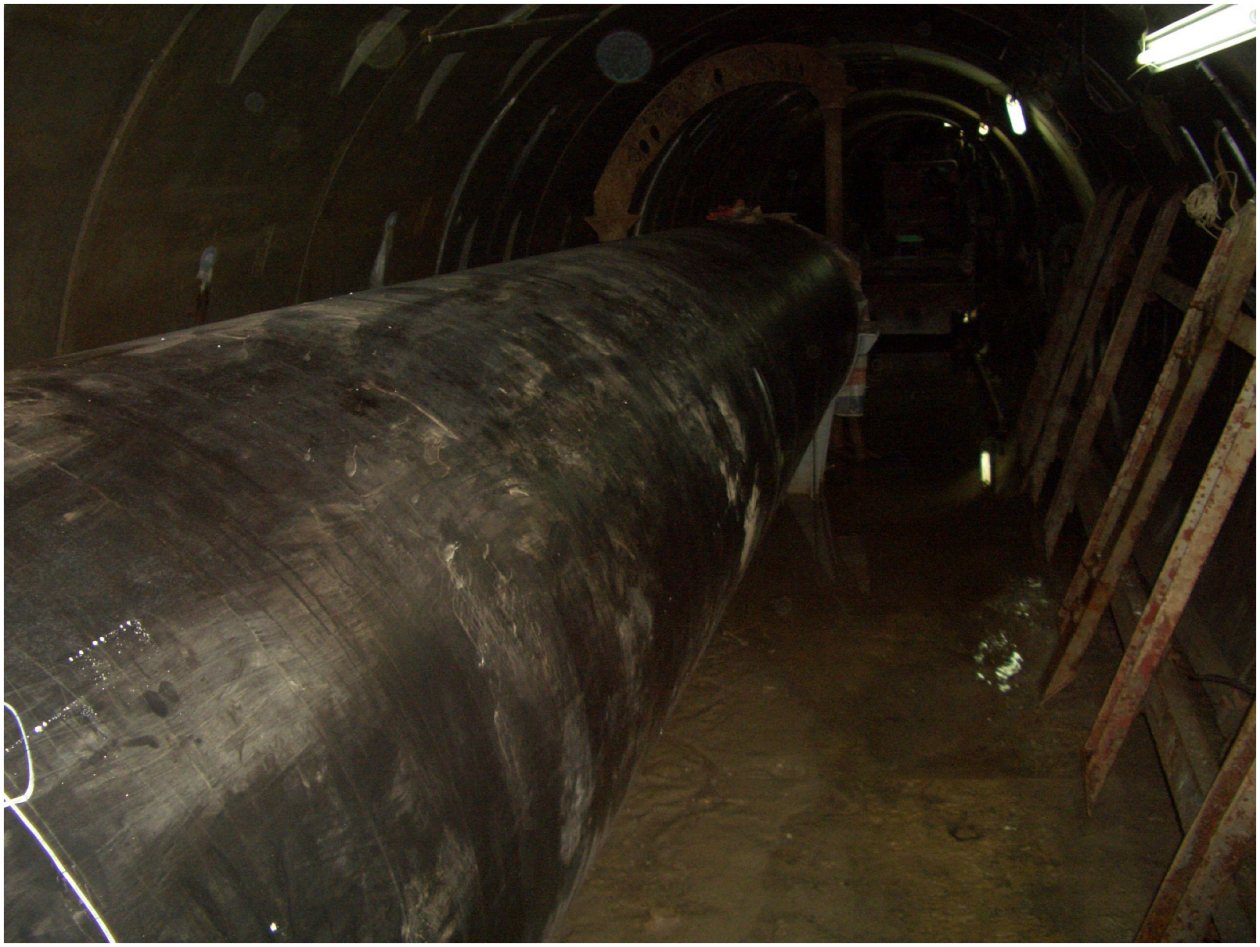
Pipelines.

**Fig 20:**
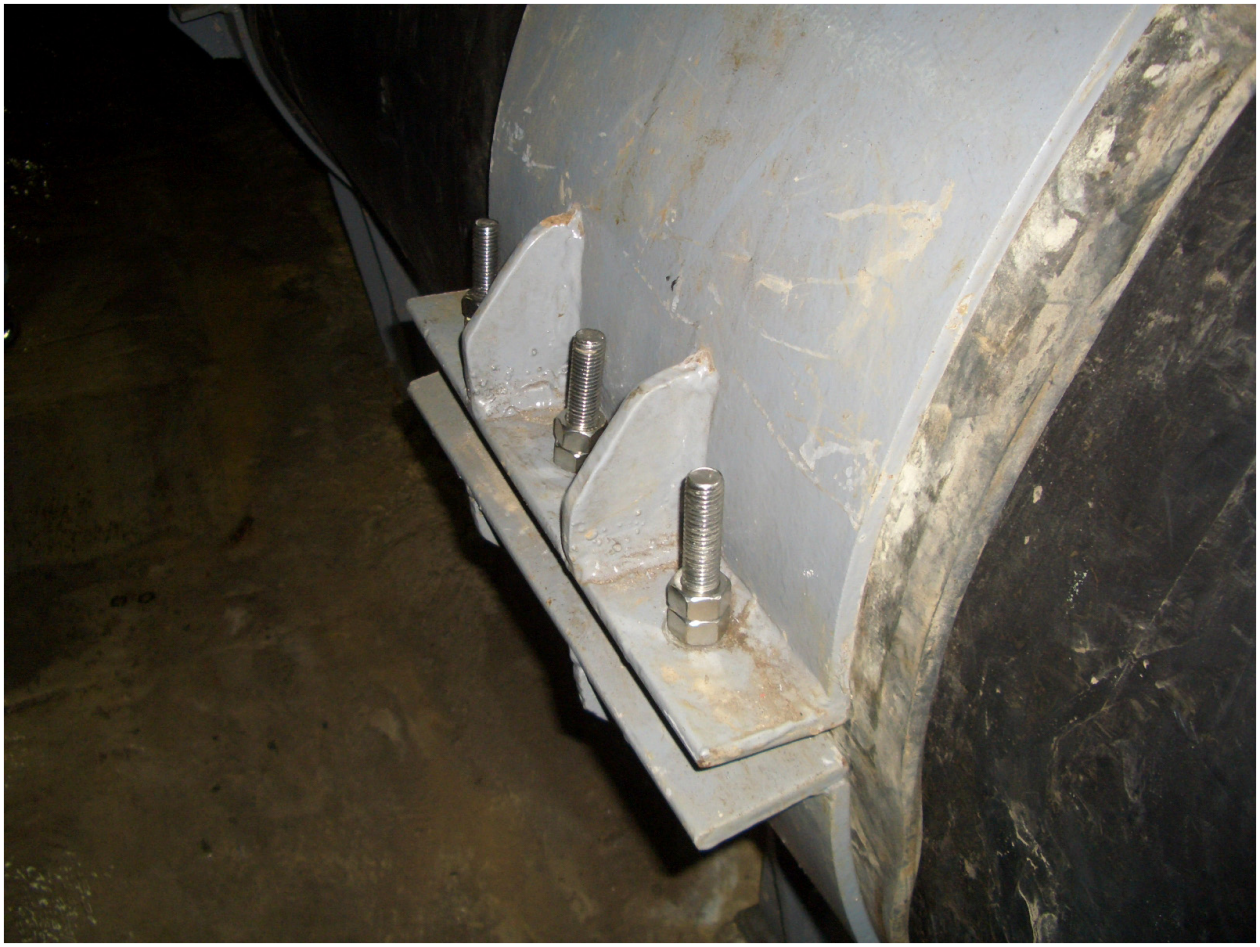
Fixed pipelines.

**Fig 21:**
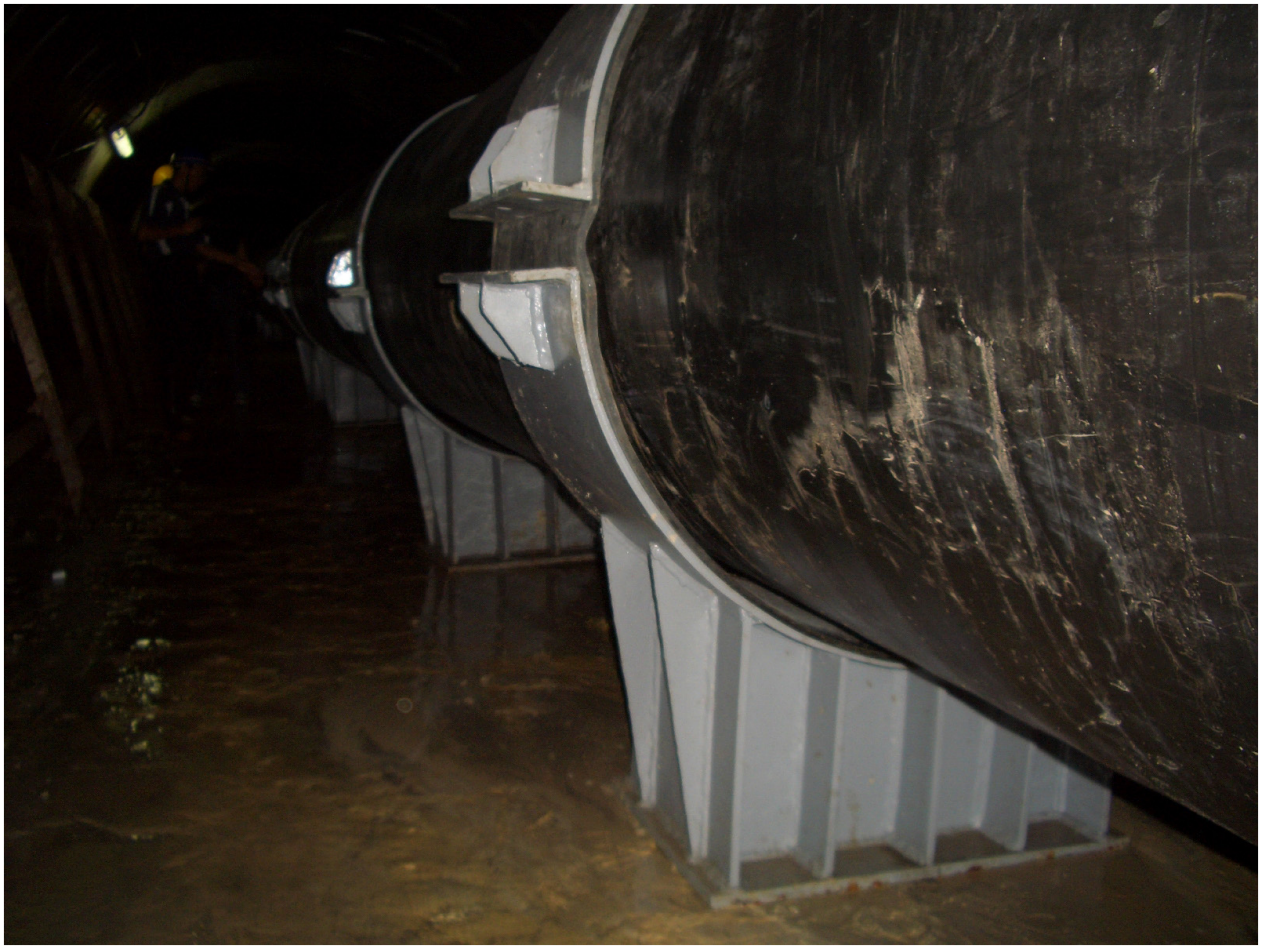
Unfixed pipelines.

## 7. Conclusions

1. According to the actual conditions of pipeline embankment laying at tunnel entrance and exit, the statically indeterminate mechanical modal of bend for tunnel pipeline with anchor blocks subjected to vertical earth pressure and transverse horizontal earth pressure is established. By using unit load method, the axial force of the anchor block for protected pipeline is obtained.
2. The thrust acted on anchor block is calculated by the presented method in this paper for X80 steel tunnel pipeline from West-East Gas Transmission Pipeline Project in China. The analytical results are compared with finite element results (by CAESAR, AUTOPIPE, PIPEPAK, ANSYS software), and the maximum relative error is only 4.1%.
3. A code of pipeline design was developed using the method presented. Meanwhile, it has been used in the West—East Gas Project.
